# Ninety Years Old

**Published:** 1874-10

**Authors:** 


### HALL'S

### JOURNAL OF HEALTH

AND

### MISCELLANY.

VOL. XXI.—OCTOBER, 1874.—NO. X.

### NINETY YEARS OLD,

WITH a good appetite three times a day, delicious sleep, and not an ache or a pain in the whole body, the mind all the while fully alive to what is going on in the world, and all the time in good spirits. This is said of Ex-Gov. Throup of New York. He retires at nine and rises at six, taking a nap in the forenoon and sometimes in the afternoon also; takes breakfast at eight, dinner at one, and tea at sundown. In suitable weather he spends a greater part of the forenoon in his garden, directing his men and assisting them, and for a short time in the afternoon he is employed in the same way. He uses no spirituous liquors, but takes claret wine every day at dinner.

There are three things in the above narration which, if persistently carried out in early life, would do more than all others towards giving all an enjoyable old age.

Regularity in eating.

Abundant sleep.

A large daily exposure to out-door air.

Regularity in eating, either two or three times a day, with nothing whatever between meals, not an atom of anything, would almost banish dyspepsia in a single generation; as frequent eating is the cause of it in almost all cases, especially if irregular and fast. Abundant sleep and rest from childhood make nervous diseases a rarity; to insufficiency of regular sleep and insufficiency of rest may well be attributed nine-tenths of all sudden deaths, and a premature wearing out, before the age of sixty years. All hard workers, whether of body or brain, ought to be in bed nine hours out of the twenty-four, not that so much sleep is required, but rest, after the sleep is over; every observant reader knows how the system yearns for rest in bed after a good sleep, and it is a positive gain of energy to indulge in it. Every hour that a man is out of doors is a positive gain of life, if not in a condition of chilliness, because no in-door air is pure; but pure air is the natural and essential food of the lungs, and the purifier of the bood, the want of which purification is the cause or attendant of every disease, while every malady is alleviated or cured by an abundant exposure to out-door air. If city wives and daughters would average two or three hours every day in active walking in the open air, it would largely add to exemption from debility, sickness and disease, and would materially add to domestic enjoyment and the average duration of life.

